# Multi-Level Opinion Dynamics under Bounded Confidence

**DOI:** 10.1371/journal.pone.0043507

**Published:** 2012-09-19

**Authors:** Gang Kou, Yiyi Zhao, Yi Peng, Yong Shi

**Affiliations:** 1 School of Management and Economics, University of Electronic Science and Technology of China, Chengdu, China; 2 Research Center on Fictitious Economy and Data Sciences, Chinese Academy of Sciences, Beijing, China; 3 College of Information Science & Technology, University of Nebraska at Omaha, Omaha, Nebraska, United States of America; Umeå University, Sweden

## Abstract

Opinion dynamics focuses on the opinion evolution in a social community. Recently, some models of continuous opinion dynamics under bounded confidence were proposed by Deffuant and Krause, et al. In the literature, agents were generally assumed to have a homogeneous confidence level. This paper proposes an extended model for a group of agents with heterogeneous confidence levels. First, a social differentiation theory is introduced and a social group is divided into opinion subgroups with distinct confidence levels. Second, a multi-level heterogeneous opinion formation model is formulated under the framework of bounded confidence. Finally, computer simulations are conducted to study the collective opinion evolution, focusing on three key factors: the fractions of heterogeneous agents, the initial opinions, and the group size. The simulation results demonstrate that the number of final opinions depends on the fraction of close-minded agents when the group size and the initial opinions are fixed; the final opinions converge more easily when the initial opinions are closer; and the number of final opinions can be approximately modeled by a linear increasing function of the group size and the increasing rate is the fraction of close-minded agents.

## Introduction

In March 2011, the panic buying of iodized salt and iodine pills was triggered in Canada, China, Russia's Far East, and United States after the Fukushima Daiichi nuclear accident. It was mostly caused by the rumor that iodized salt and iodine tablets could help ward off radiation poisoning, despite the government and health officials' statements that potassium iodide is not anti-radiation. The underlying mechanism of such process can be explained by opinion dynamics theory, which analyzes how individuals choose a convention, make a decision, schedule tasks, and put into actions.

When people confront a social issue in a given community, they will express their opinions spontaneously or unconsciously and may have different responses. In the interaction between two individuals, each can influence the other and gradually form common opinions by ignoring minority opinions and allowing opinion differences. Because the human opinion propagation is an outcome of multistage physiological and psychological processes, it is challenging to collect the dynamics of individual consciousness and define the way of an individual interacts with others. Opinion dynamics, as a macroscopic collective social phenomenon, has been a popular research topic in physics, mathematics, social psychology, computer science, anthropology, and management science.

Some physical models have been developed to explore the underlying mechanisms of opinion dynamics, even though it is extremely difficult to describe and evaluate the collective behaviors involving human emotional and psychological factors. Social network analysis and dynamical systems theory are widely applied to model the opinion evolution for a group of agents [1], [2], [3]. Two types of models, agent-based model for a finite population of agents and density-based model for an infinite number of agents with a density function on the opinion space, have been proposed to describe the collective opinion evolution [4].

In an agent-based model of opinion dynamics, each agent has an opinion described by a variable which can change in time. In the discrete case, binary values are selected to represent yes or no. However, there are situations that the opinion of an agent can vary smoothly between the extremes. For example, in the Fukushima-triggered panic buying, the attitude of an agent is not restricted to completely believe/disbelieve the rumor, but is in the range of a bounded opinion domain. Such a case is often referred to as continuous opinion dynamics [5]. Accordingly, opinion dynamics models can be classified into discrete opinion dynamics model and continuous opinion dynamics model. The Sznajd model [6], the voter model [7], and the Galam majority-rule model [8] are classical examples of discrete opinion dynamics models. Among continuous opinion dynamics models, two models under bounded confidence presented by Deffuant and Weisbuch (DW model) [9] and Hegselmann and Krause (HK model) [5], [10] have received significant attention. In the bounded confidence models, an agent only interacts with those whose opinion is close to its own under a given confidence level. In many current models, agents' opinions are described by scalars. However, opinions are comprised with multiple factors. Multi-dimensional opinion dynamics models, such as Axelrod's cultural diffusion model [11], [12], are applied to investigate such an opinion formation problem [11], [13].

Most opinion dynamics models assume that each agent is homogeneous and has the same confidence level. However, due to complex physiological or psychological factors, each social agent may have diverse confidence levels. In such cases, a heterogeneous bounded confidence model is more appropriate for the opinion evolution with agent-dependent confidence levels. The heterogeneous HK model was reformulated as an interactive Markov chain in [14]. A heterogeneous DW model and HK model were proposed for opinion evolution with agent-based version and density-based version in [4] and [15]. The agents were classified into essential and inessential, or close-minded and open-minded, according to the confidence levels. The effects of heterogeneous confidence bounds were analyzed by a series of experiments. In [16], a heterogeneous HK model was employed to give a theoretical convergence analysis under some assumptions on the existence of the equilibrium opinion vector and the time-invariant interaction topology.

When social agents are heterogeneous, the agents can be divided into fractions according to the confidence levels in the collective opinion dynamics. The fractions of heterogeneous agents, essentially the heterogeneous confidence levels, will influence the communication network and evolutions of the opinions. In addition to the fractions of heterogeneous agents, both the initial opinions and the group size also play a key role in the collective opinion evolution, which have been analyzed in the homogeneous bounded confidence models. Existing studies in homogeneous bounded confidence models have shown that the group size will not decide the number of the final opinions. However, it is still an open problem in the heterogeneous cases. Additionally, in literatures, it is assumed that the initial opinions are uniformly distributed in both homogeneous and heterogeneous bounded confidence models. However, the initial opinions of social agents are often non-uniformly distributed in real world situations. The objective of this paper is to build a heterogeneous opinion dynamics model and study the influence of the three factors, heterogeneous fractions, non-uniformly distributed initial opinions and group size, on the convergence of the opinion dynamics. We firstly differentiate the social group into multiple levels of subgroups according to the individual confidence levels. The sizes of the subgroups need not be same, an extended heterogeneous HK model is then built for the differentiated group. Then a series of simulation examples are used to investigate the influences of the three key factors on the collective opinion convergence.

The rest of the paper is organized as follows. Section 1 describes the bounded confidence models, including the homogeneous HK model and the proposed extended opinion dynamics model with heterogeneous confidence levels. Section 2 presents details of simulations, which are designed to study the impacts of three key factors on the final opinions of the heterogeneous opinion dynamics. Section 3 concludes the paper.

## Methods

### Bounded confidence models

Theoretically, each agent can interact with every other agent, regardless of their opinions. However, in practice, agents interact with each other only if their opinions are sufficiently close to each other, a situation referred as bounded confidence (BC). According to bounded confidence, two agents must not have significantly different opinions in order to build up a successful interaction. In physics, it is well known that if two particles are too far apart, they do not exert any influence on each other. Thus, bounded confidence parallels somewhat the range of interaction of particles. However, the distance involved in bounded confidence is not spatial, rather, it is defined in an opinion space. For simplicity, we can assume that the opinion space is one dimensional, that is, all agents exchange their opinions which only focus on one aspect of a specific issue. For example, in the Fukushima-triggered panic buying case, all agents only concern about whether the iodized salt and iodine tablets could help prevent the human body from absorbing radioactive materials.

In a bounded confidence model of a network of multiple autonomous agents, every agent is initially assigned a random opinion described by a real value within a given opinion space. Mathematical models of opinion dynamics under bounded confidence have been developed by Deffuant and Weisbuch (DW model) and by Hegselmann and Krause (HK model). In contrast to discrete opinion dynamics, all agents may start with different continuous opinions, and the possible interaction scenarios are more complex. The opinion clusters emerge in the final stationary opinion state. The opinion clusters could be one (consensus), two (polarization), or more (fragmentation).

In the DW model, two randomly selected agents update their opinions at any given time step. If their opinions differ by more than certain confidence level, their opinions remain unchanged; otherwise, each agent moves to a new opinion which is an arithmetic average of its previous opinion and that of the other agent. In the HK model, agents synchronously update their opinions by averaging all opinions in their confidence bounds. The DW model is suitable to describe the opinion dynamics of traditional exchange way, where people meet in small groups and communicate face-to-face. In contrast, the HK model is intended to describe an effective interaction involving many people at the same time. With the development of information technology, synchronous exchange of opinions of a large number of agents can be easily achieved. Therefore, this study concentrates on bounded confidence opinion dynamics under the framework of the HK model.

### Homogeneous HK model

Consider a social group having 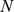 agents. Each agent 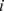 at time 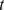 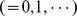 has a continuously varying opinion state 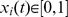. In the Fukushima-triggered panic buying, the state variable 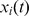 represents the attitude of looking upon the rumor. If the 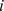
th agent completely believe the rumor, 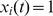; if he does not believe it at all, 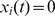. However, if agent 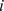 is not sure the iodized salt and the iodine pills can prevent the human body from radiation injuries, the opinion may range from 0 to 1. The initial opinion 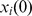 follows uniformly random distribution in 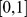. In the HK model, the opinions of agents influence each other when they are smaller than a given confidence level, that is, agent 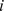 only takes agent 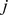 into account if the difference of their opinions 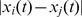 is less than a certain confidence level of agent 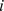. Specifically, the HK model in [5] and [10] was given by
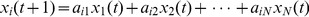
(1)where 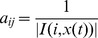 denotes the weight of the neighboring agent 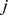 which can influence the opinion of agent 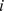. The set 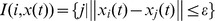 is the opinion neighbor set whose opinion difference with agent 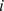 is not greater than the confidence level 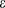. The symbol 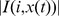 denotes the number of opinion neighbors of agent 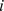. Because 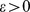 is the given confidence level and it is the same for all agents, the HK model is called homogeneous. The vector 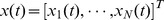 denotes the group opinion profile. The HK model indicates that the opinion updates of agents depend on the arithmetical mean value of neighbors' opinions in the multi-agent social group considered.

**Figure 1:**
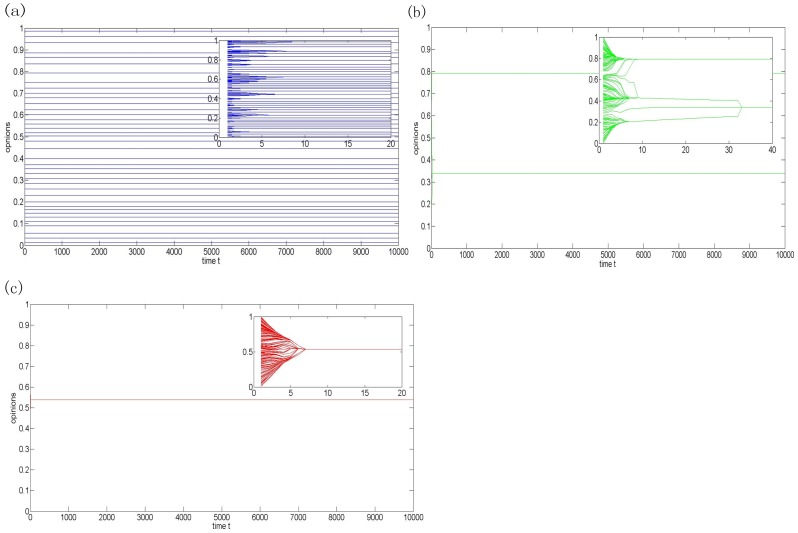
Plot of opinion evolution of model (1) with N = 200. (**a**)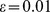; (**b**)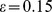; (**c**)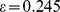. The blue, green, and red lines denote the opinions of agents with low, middle, and high confidence levels, respectively.

A set of simulations is given to illustrate the evolution of the collective opinions with homogeneous confidence level for HK model (1), which will be compared with the heterogeneous HK model proposed in the subsequent part. Suppose there is a social group with 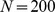. The running time is set as 10,000 in the homogeneous HK model. Assume the initial opinions take values in 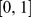 uniformly. Figure 1 shows three patterns of the final opinion profiles under corresponding homogeneous confidence levels. The x-axis represents simulation time step and the y-axis represents the evolutions of opinions. For a group size 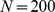, it takes less than 10 steps to reach a stable pattern. When the 200 agents are close-minded and have a very small confidence level 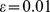,,the final opinions are fragmental and form 39 distinct final opinions (See Figure 1a). When the homogeneous confidence level 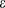 increases to 0.15, the number of the final opinions decreases to 2 and a polarization pattern emerges (Figure 1b). Finally, the 200 agents easily reach consensus as time goes when 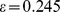 (Figure 1c). The three simulation results show that, when all agents in a given society have the same confidence level, the larger the confidence, the higher the probability of reaching consensus.

In the HK model, there is an assumption that each agent has a homogeneous confidence level. A large number of simulation results under the hypothesis have indicated that the number of the final opinion clusters only depends on the homogeneous confidence level 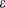. We can observe from Figure 1 that the number of final opinion clusters is a non-increasing function of 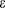. In particular, there exists a lower bound 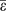 for a given social group such that for all 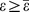 and an arbitrary initial opinion, the final opinion profile will reach consensus. However, the hypothesis of homogeneous confidence level is somewhat restricted in real world situations.

## Discussion

In reality, agents in a social group may have heterogeneous confidence levels. For example, distinct opinions on the radiation leaks of the Fukushima nuclear plant appeared among people with different genders, ages, or educational degrees, etc. It is reasonable to assume that agents have heterogeneous confidence levels and also important to investigate how the collective opinions evolve under such an assumption and address some open problems. For example, how do agents update their opinions when they have heterogeneous confidence levels? What is the state of the final collective opinion under such case? Which factors affect the evolution of the collective opinions?

### Social differentiation in opinion dynamics

In the social life of human beings, each individual always belongs to some groups, which share similar characteristics, interests, rank, and so on. The human society can be differentiated into different groups according to various criteria. For example, the social group can be divided into upper class, moderate class, and lower class by power, status, or wealth; huge, large, and small group by size; family, friends, and strangers by the degree of intimacy. A society can be differentiated vertically or horizontally into subgroups based on different interests and needs of its members [17], [18], [19], [20]. Group members can be heterogeneous in terms of their position in the vertical hierarchy or in the horizontal division. Groups may also have multiple subgroups whose goals and desires have varying overlap with the overall group's goals and desires.

Some works focused on opinion formation based on a vertical differentiation, such as opinion leaders in [21], authority agents in [22], and informed agents in [23]. In this paper, only horizontal differentiation is adopted to a given society according to the heterogeneity of the confidence levels. For example, in the Fukushima-triggered panic buying, an agent may have a high or low confidence level on the opinion that iodized salt and iodine pills could be anti-radiation, regardless of the social status or position (a vertical differentiation). Thus it is reasonable to differentiate agents into some opinion subgroups according to their different confidence levels (a horizontal differentiation) in the investigation of the collective opinion dynamics [24]. Under such a differentiation, agents with low confidence levels may not join the panic buying, while agents with larger confidence levels have a higher probability to join the collective buying [25].

**Figure 2:**
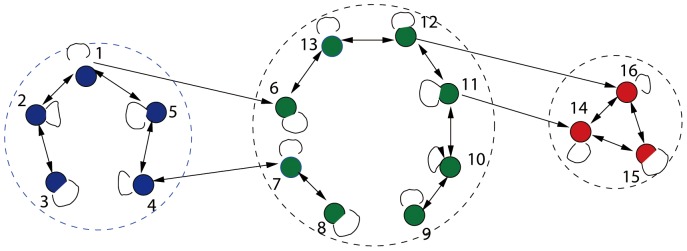
A social system with three opinion subgroups (the confidence levels 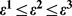 from left to right).

Consider a social system with 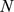
agents which have 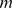
heterogeneous confidence levels. Now let us differentiate the social system according to the agent's confidence level. Then the multi-agent group can be divided into 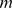 opinion subgroups which have confidence levels 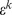 for 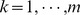, respectively. Agents with confidence level 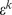 belong to the 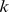
'th subgroup. Without loss of generality, assume that the confidence levels 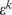 ∈ 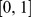. For example, when the social system have three heterogeneous confidence levels, then three opinion subgroups can be illustrated in Figure 2, where three opinion subgroups are illustrated by dashed circles. The colors blue, green and red, respectively, denote the low, moderate and high levels.

If there exists an arc starting from node 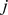 and ending at node 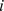, we assign a positive number 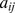 as the weight of the arc. For simplicity, assume the edges in each same subgroup are bidirectional, while the edges between two different subgroups may be unidirectional. As shown in Figure 2, there are four edges among three subgroups, i.e., the links between node 1 and 6, 4 and 7, 11 and 14, 12 and 16, three edges of them are unidirectional. In the sense of confidence levels, the weight 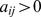 means that agent 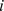 can accept the opinion of agent 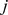. For example, 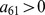 and 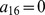, which indicate that agent 6 can use the opinion of agent 1 but agent 1 ignores the opinion of agent 6. Additionally, the size of each subgroup may not be uniformly distributed. In general, the sizes of subgroups with the smallest and largest confidence levels are comparatively small.

### Multi-level opinion model

Let a social network has 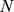 agents. Each with its own opinion shows the degree of adopting or rejecting a certain behavior. Suppose the whole opinion space is 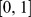, where 1 represents “complete accept”, 0 represents “absolute reject”, and the values between 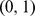 represent the fuzzy levels of opinion. Let 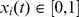 denotes the opinion of agent 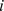 at time 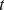.The n-dimensional vector 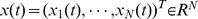 denotes an opinion profile, which is a group opinion and aggregates all the private opinions.

For agent 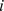 belonging to subgroup 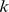, if the opinion of agent 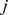 satisfies 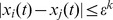 for some 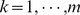, agent 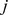 is called a neighbor of agent 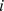. During the evolution of the collective opinions, at each time step, each agent 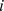 firstly searches his neighbors according to his own confidence level. Then, agent 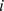 updates his opinion according to the following rule
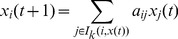
(2)where the set 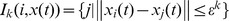 is the neighbor set of agent 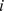 with confidence level 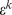 at time t, 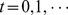. The positive number 
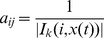
 is the opinion weight of the neighbor 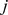 on agent 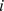. Thus, the update rule (2) is essentially a weighted average algorithm, which assumes all neighbors play the same roles in deciding the new opinion of agent 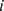. Although model (2) has a similar form with the homogeneous HK model, it in fact consists of 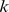 subsystems.

It is noted that the weights 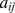 and 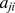 are not necessarily equal in the proposed heterogeneous opinion dynamics (2). The definition of 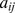 implies that it is a time-varying function depending on time t, the confidence levels 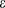 and the initial opinions. From Figure 2, if agent i and agent j belong to different opinion subgroups, even though there is a link from agent i to agent j, there may be no link from agent j to agent i. thus, 
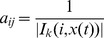
 while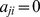. Even if agent i and agent j are with a same subgroup, their neighbors may come from distinct subgroups and the neighbor numbers may be different.

## Results

In this section, we investigate the impacts of the three key factors: the fractions of heterogeneous agents, the non-uniformly distributed initial opinions and the group size, on the extended opinion formation model (2) by a series of computer simulations. Firstly, the impact of the heterogeneous confidence levels on the opinion dynamics is considered. Due to the fact that agents within a social group generally have heterogeneous confidence levels, without loss of generality, the group can be divided into opinion subgroups such that each subgroup possessed one confidence level. Thus, agents with same confidence levels are belonging to a same subgroup. Secondly, since the initial opinions play a key role in convergence of the opinion dynamics, we will focus on the impact of non-uniformly distributed initial opinions on the evolution of the collective opinions for the heterogeneous subgroups. Finally, an exploration is attempted to investigate the impact of the group size on the collective opinion evolution.

### Impacts of the fractions of agents in different groups

The whole social group with 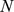 agents is differentiated according to the magnitudes of confidence levels. The heterogeneity of confidence levels exists in all societies during a public opinion formation process. For example, the Fukushima nuclear disaster created a nuclear radiation fear across the Asian pacific region. The society showed different attitudes on some spreading opinions about nuclear radiation. If individuals have no nuclear related knowledge, they tend to adopt the spreading opinions easily,such as using salt to prevent radiation, and their confidence levels on this issue are higher than those are knowledgeable about nuclear radiation. At the same time, the differentiated opinion subgroups evolve according to the proposed rule (2).

Suppose that the social group consists of 200 agents and is divided into three opinion subgroups according to the confidence levels. Agents in the three subgroups have confidence levels 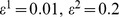, and 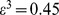, respectively. The three subgroups are called close-minded (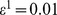), moderate-minded (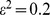), and open-minded (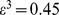). The size of each subgroup is not uniformly distributed. In reality, the number of agents with the lowest or highest confidence levels is comparatively less than those with middle confidence levels. Most people are uncertain during the opinion propagation and can partly accept the opinions of the others depending on their initial opinions and the update rule (2).

At the beginning, the fractions of agents in the three subgroups are arbitrarily set as 1%, 79%, and 20%, respectively. Then the fraction of close-minded agents in the three subgroups increases to 2%, 5%, and 10%. Finally, the fraction of close-minded agents is fixed as 10%, and the fractions of open-minded agents are set as 20%, 40%, and 70% in three simulations. The initial opinions of the 200 agents are uniformly distributed in the opinion interval [0,1]. Furthermore, to better illustrate the impacts of the heterogeneous fractions on the collective opinion evolution, the initial opinions of the 200 agents are fixed in all simulations [26].

**Figure 3:**
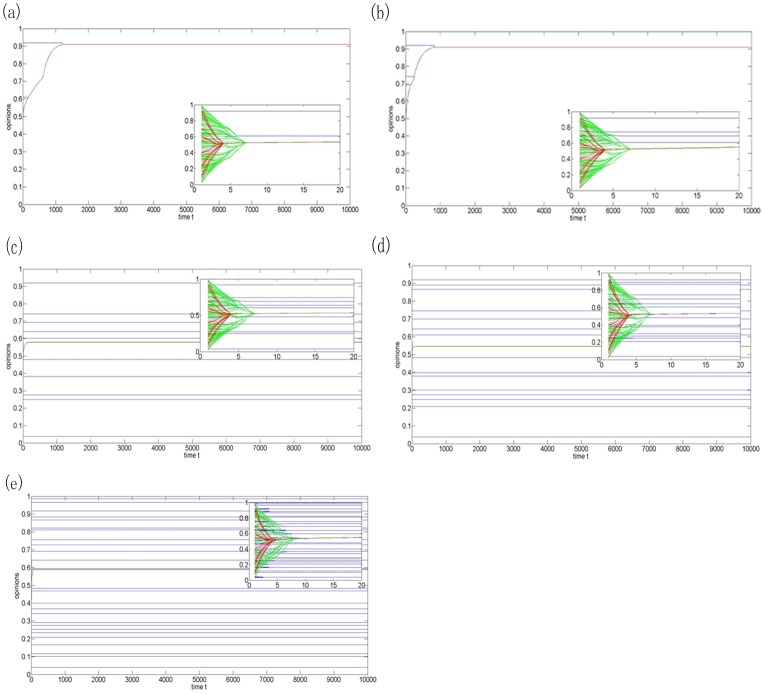
Plot of opinion evolution of model (2) with N = 200, 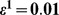 (blue-for close-minded agents), 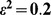 (green-for moderate-minded agents), 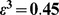 (red-for open-minded agents). (a) The fractions of agents in three groups are, respectively, 1%, 79%, and 20%; (b) The fractions of agents in three groups are, respectively, 2%, 78%, and 20%; (c) The fractions of agents in three groups are, respectively, 5%, 75%, and 20%; (d)The fractions of agents in three groups are, respectively, 10%, 70%, and 20%; (e) The fractions of agents in three groups are, respectively, 30%, 50%, and 20%.

Figure 3a and 3b show that the group can reach consensus when the extremists are two or four agents. Even though the close-minded agents have very low confidence level 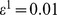, they can be affected by the moderate-minded agents, which are the majority of the group. Before time step 400, the close-minded agents nearly keep parallel opinions due to the large initial opinion difference. However, there is a jump around time step 1200 and the two final opinions led by the close-minded agents merge together, which indicates that there is less influence of the extremists on the collective opinion when the fractions of the close-minded agents are 1% and 2%. In the homogeneous counterpart (See Figure 1c), 200 agents (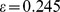) reach consensus around no less than 10 time steps. Note that the average confidence levels in Figure 3a and 3b are greater than 0.245, but the convergence rates are slower than that the homogeneous situation, which implies that it is more difficult to reach consensus for a social group having a certain number of close-minded agents. In addition, the final opinion approaches to the close-minded agent whose initial opinion is far away from the average initial value of all agents.

As the fraction of close-minded agents increases from 2% to 5% (Figure 3c), the numbers of close-minded agents turn to 10 and 11 final opinion clusters form finally. In the initial stage, the opinions of the close-minded agents are unchanged, while the moderate-minded and open-minded agents quickly form polarity and converge to one final opinion by taking into account their similar initial opinions.

**Figure 4:**
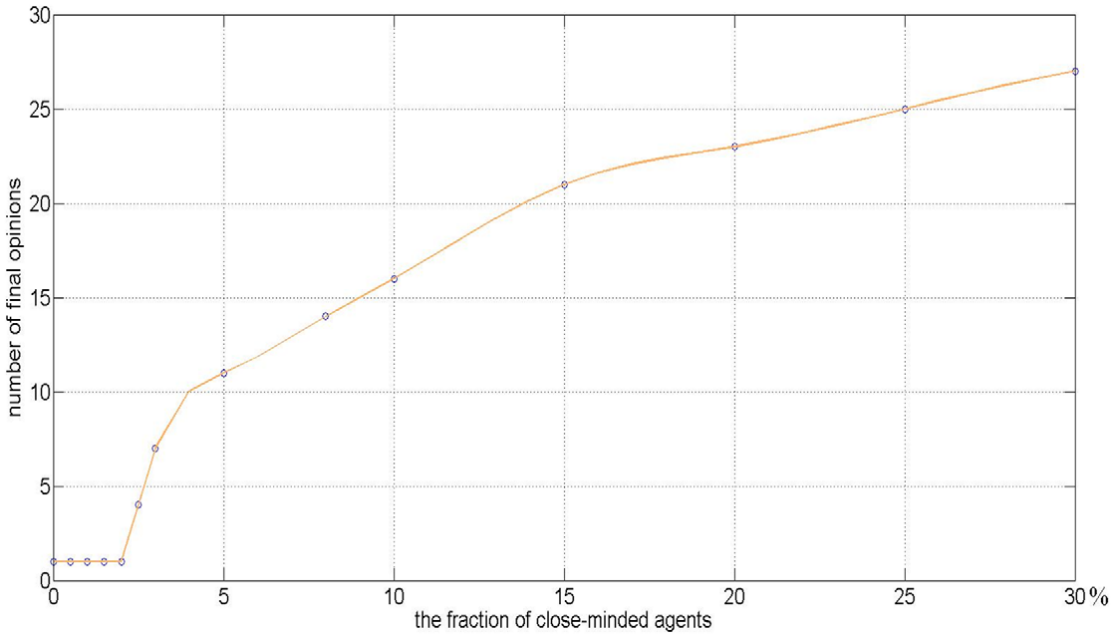
The relationship between the number of final opinions and the fraction of close-minded agents.

The fraction of the close-minded agents increases to 10% in Figure 3d and 30% in Figure 3e, which aggravates the final opinions' fragmentation. As shown in Figure 3a, 3b, 3c, 3d, and 3e, the number of final opinion clusters increases as the fraction of close-minded agents increases. Although the close-minded agents belong to the same confidence level subgroup, most of them maintain their own initial opinions due to the distinct difference of their initial opinions and their small confidence levels. As shown in Figure 4, there exists a critical value 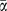 of the fraction of close-minded agents to reach consensus in a given society. When the group size is 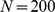, the critical number is 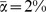. Once the fraction of the close-minded agents is bigger than the critical number, the final opinions will be fragmental. Denote 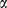 and 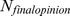 as the fraction of close-minded agents and the number of final opinion clusters, respectively. Thus, in this example, a linear model can be setup as follows.
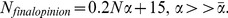
(3)


**Figure 5:**
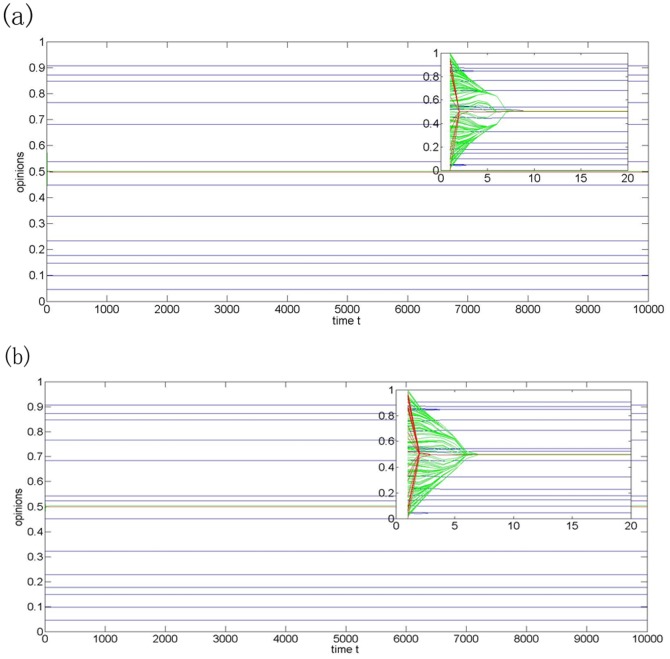
Opinion evolution of model (2) with N = 200, 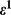 = 0.01(blue-for close-minded agents), 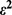 = 0.2(green-for moderate-minded agents), 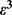 = 0.45(red-for open-minded agents). (a)The fractions of agents in three groups are, respectively, 10%, 50%, and 40%; (b) The fractions of agents in three groups are, respectively, 10%, 20%, and 70%.

To further study the impacts of the fractions of agents in different subgroups on the collective opinions, the fraction of open-minded agents increases from 20% to 40% and 70%, while the fraction of close-minded agents is fixed at 10%. The numbers of final opinion clusters are similar in Figure 3d, Figures 5a, and 5b, but the increase in the number of open-minded agents leads to a quick convergence rate of the opinion evolution of moderate-minded and open-minded agents.

Figure 5 shows that it is impossible to eliminate the fragmentation of the collective opinions by increasing the fraction of open-minded agents. The results on the collective opinion evolution under the heterogeneous model (2) are different from those under the homogeneous HK model. Figures 3 to 5 reveal that the fraction of the close-minded agents decides the number of the final opinions, which can also be found in the Fukushima panic buying. In a fixed size community with large fraction of open-minded agents, even the overwhelming majority involved in the panic buying but there are a few people insisting their resistance altitudes. In summary, if the social agents belong to several distinct opinion subgroups and their opinions updates are determined by the heterogeneous HK model (2), then there is a linear increasing relationship between the number of final opinions and the fraction of close-minded agents, while the convergence rate of the final opinions depends on the fraction of open-minded agents.

### Impacts of the initial opinions on the opinion dynamics

In this section, the influence of the second factor on the collective opinion evolution is analyzed. As mentioned in [4], [14], [15] and also shown in Figure 3 and Figure 5, the evolution of the collective opinion dynamics depends on the initial opinions and the confidence levels. How do agents behave themselves in the Fukushima-triggered panic buying when they have different initial opinions on the rumor? Most agents cannot distinguish whether the spreading saying is true or false because of the fear of radiation damage and the lack of knowledge and experience about the matter. Generally, agents that have the similar initial opinion on the spreading saying will easily form an opinion sub-group. Thus, it is reasonable to assume that a certain community may have similar initial opinions. In this case, it is important to answer how the collective opinion evolves when individuals have a small initial opinion difference. In this example, the initial opinions of agents are always fixed in the opinion interval [0.5,0.55].

**Figure 6:**
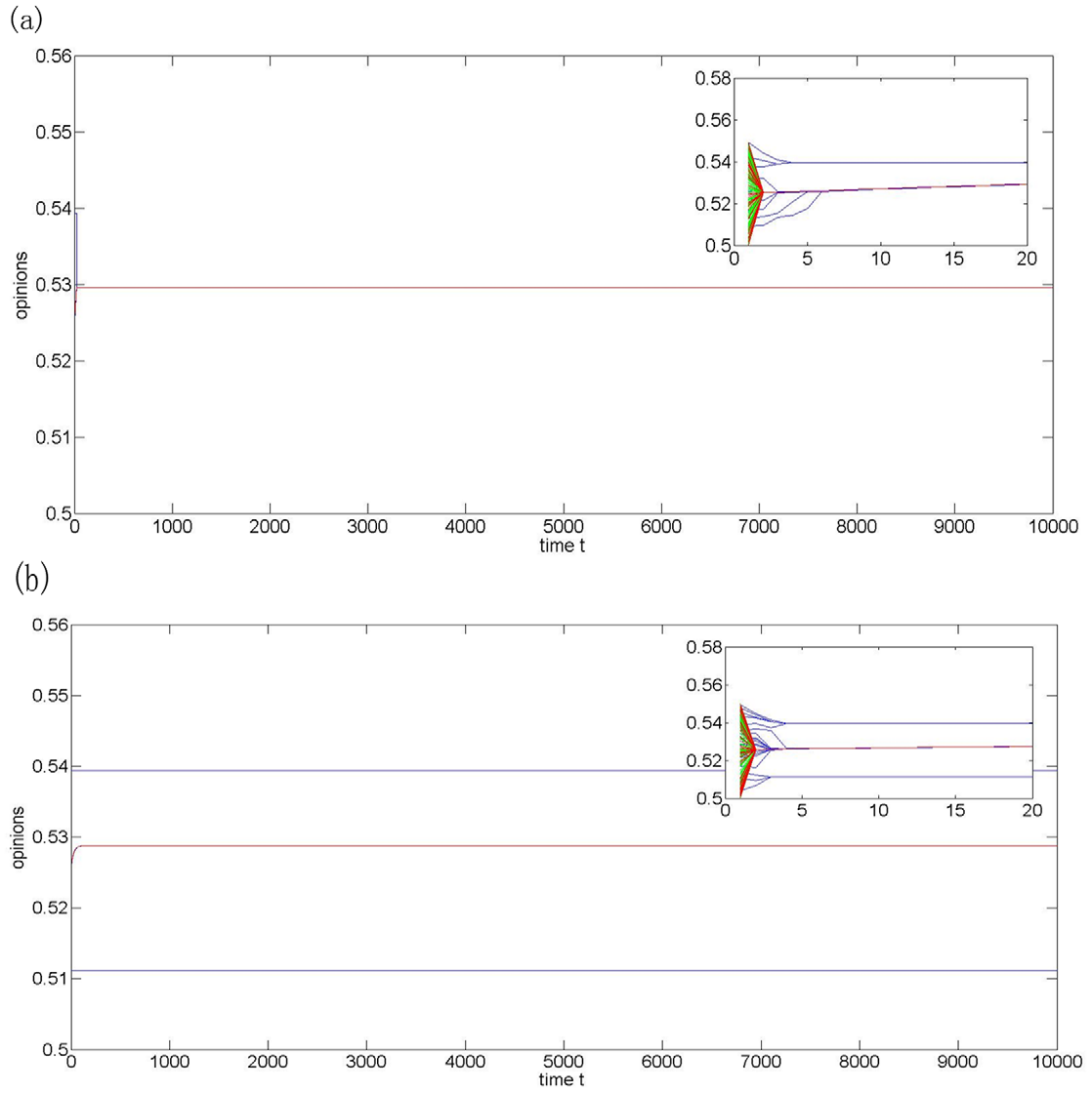
Opinion evolution of model (2) with N = 200, 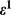 = 0.01 (blue-for close-minded agents), 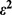 = 0.2 (green-for moderate-minded agents), 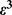 = 0.45 (red-for open-minded agents) and the initial opinion space [0.5,0.55]. (a) The fractions of agents in three groups are, respectively, 5%,70%, and 25%; (b) The fractions of agents in three groups are, respectively, 10%, 70%, and 20%.

Though there are still 10 close-minded agents, the agents reach consensus due to their small differences in the initial opinions (see Figure 6(a)). Figure 6(b) shows that the opinion fragment is unavoidable when the number of close-minded agents becomes large enough, even though the initial opinions are very close. The simulation results are consistent with the reality. For instance, in the panic buying case, more final opinion clusters will be formed if the fraction of close-minded agents increases in the fixed-size group.

Note that the result in this section is a further investigation of the relationship between the initial and final opinions, which has been studied in the literature [4], [5], [10], [15]. Different from previous studies, this research assumes that agents have non-uniformly distributed initial opinions.

We can see from the simulations above that, if the number of close-minded agents is the same and the opinion update rule is determined by the heterogeneous BC model (2), the probability of reaching consensus will increase as the differences of the agents' initial opinions decrease. The possibility of the fragmentation of collective opinions will increase as the fraction of close-minded agents increases in a fixed-size group.

### Impacts of the group size on the opinion dynamics

The last factor under consideration is the group size of the social community. Both the homogeneous Deffuant model [9] and HK model [5] [10] concluded that the final collective opinions will reach consensus if the confidence level of agents is larger than a certain threshold, regardless of the size of the social group. Will this also be true for heterogeneous case?

To make a contrastive analysis, the proposed heterogeneous model (2) is simulated by taking the group size N = 100, 200, 500, 1000, respectively. The confidence levels of three subgroups are 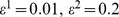 and 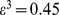, respectively. The fractions of agents of the three subgroups are 5%, 75%, and 20%.

**Figure 7:**
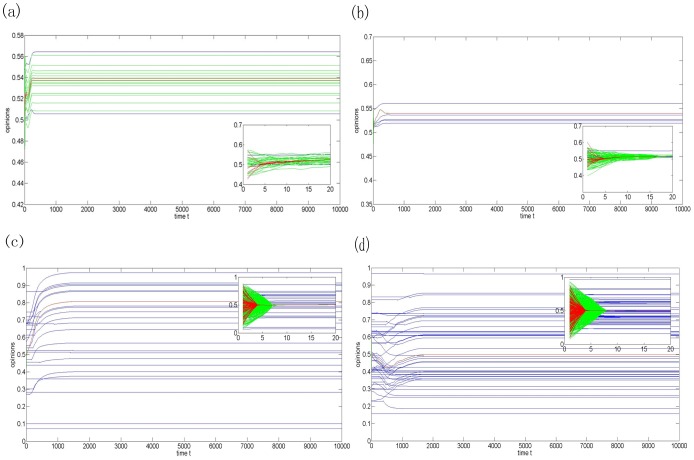
Opinion evolution with different group size (a) N = 40 (b) N = 100 (c) N = 500 (d) N = 1000 (blue-for close-minded agents; green-for moderate-minded agents; red-for open-minded agents).

In Figure 7(a), when the social group size *N* = 40, there are 17 final opinions. When the group size increases to 100, the number of final opinions declines to 6 (Figure 7(b)). As the group size further increases, the number of final opinions increases with some constant rates as shown in Figure 7(c) and 7(e).

**Figure 8:**
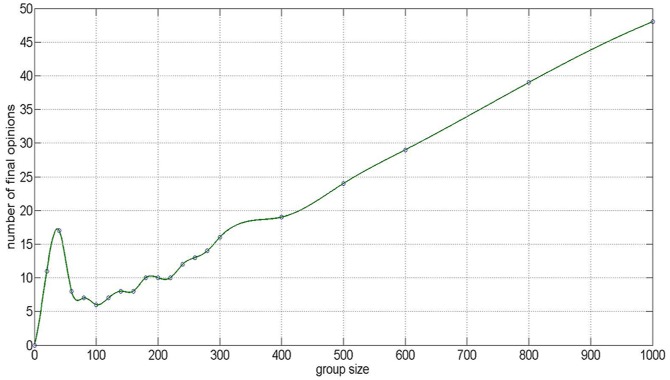
The relationship between the number of final opinions and the group size.

To obtain a systemic investigation of the relationship between the number of final opinions and the group size, numerical experiments have been done for the opinion evolution with the group size *N* = 20, 40, 60, 80, 100, 120, 140, 160, 180, 200, 220, 240, 260, 300, 400, 500, 600, 800, and 1000 (See Figure 8). For each group size, we run the experiments 100 times and average the results as the number of the final opinions. The experiments show that the number of final opinions increases with a constant rate when the group size reaches a critical value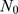. In fact, the constant rate is the fraction of close-minded agents. If 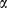 is used to denote the fraction of close-minded agents, the relationship can be modeled using the following linear equation
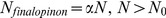
(4)


In fact, the relationship (4) can also be found for other fraction combinations of agents, for example, when the fractions of agents of the three subgroups are 1%, 79%, and 20%, the growth rate of the number of final opinions with respect to the group size will be 1%. In other words, if one wants the group to reach consensus, that is, 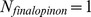, then the fraction of close-minded agents is inverse proportional to the group size.

Let's take the Fukushima-triggered panic buying as an example. In a small size community, whether the agents can reach a consensus is decided by the fraction of close-minded agents. If there are 100 people in a community, when the fraction of closed minded agents is 1%, then the people may easily to reach consensus. Once the group size increases, an agent may be more confused about whether he/she should believe spreading opinion that the iodized salt can prevent nuclear radiation. Even the fractions of heterogeneous agents is fixed, the number of close-minded agents is increasing. Then according to the simulation result (3) in Section 2.1, agents are harder to form agreement. However, for moderate-minded agents and open-minded people, it is a safe way to follow the majority and join the panic buying if only some closed-minded agents deliberately insist on the anti-radiation effect of salt.

In a word, if the opinion update rule is determined by the heterogeneous HK model (2) and the initial opinions are distributed uniformly in the opinion interval [0,1], the number of final opinions can be approximately described by a linear increasing function of the group size. Moreover, the growth rate approaches to the fraction of close-minded agents as group size goes to infinity.

## Conclusions

Under the framework of the HK model, this paper proposes an extended opinion evolution model, which is preferable for a real social group when heterogeneous confidence levels of agents are involved. The relationship between the HK model and the proposed extended model was analyzed firstly. Then three key factors of opinion convergence are investigated by using computer simulations. The first factor is the fractions of heterogeneous agents and the associated results show that the number of final opinions has a linear increasing relationship with the fraction of close-minded agents when the other two factors are fixed. The second factor is the initial opinions of agents and the simulations indicate that the collective opinions converge more easily when the initial opinions are closer. The third factor is the group size. The simulations demonstrate that the number of final opinions can be approximately modeled by a linear increasing function of the group size and the increasing rate is the fraction of close-minded agents.
